# Innovative model of delivering quality improvement education for trainees – a pilot project

**DOI:** 10.3402/meo.v20.28764

**Published:** 2015-09-22

**Authors:** Kannan Ramar, Curt W. Hale, Eugene C. Dankbar

**Affiliations:** 1 Division of Pulmonary and Critical Care, Mayo Clinic, Rochester, MN, USA; 2 Quality Improvement Advisor, Quality Academy, Mayo Clinic, Rochester, MN, USA; 3 Department of Systems and Procedures, Mayo Clinic, Rochester, MN, USA

**Keywords:** quality improvement, flipped classroom, education innovation, trainees, fellows

## Abstract

**Background:**

After incorporating quality improvement (QI) education as a required curriculum for our trainees in 2010, a need arose to readdress our didactic sessions as they were too long, difficult to schedule, and resulting in a drop in attendance. A ‘flipped classroom’ (FC) model to deliver QI education was touted to be an effective delivery method as it allows the trainees to view didactic materials on videos, on their own time, and uses the classroom to clarify concepts and employ learned tools on case-based scenarios including workshops.

**Methods:**

The Mayo Quality Academy prepared 29 videos that incorporated the previously delivered 17 weekly didactic sessions, for a total duration of 135 min. The half-day session clarified questions related to the videos, followed by case examples and a hands-on workshop on how to perform and utilize a few commonly used QI tools and methods.

**Results:**

Seven trainees participated. There was a significant improvement in knowledge as measured by pre- and post-FC model test results [improvement by 40.34% (SD 16.34), *p*<0.001]. The survey results were overall positive about the FC model with all trainees strongly agreeing that we should continue with this model to deliver QI education.

**Conclusions:**

The pilot project of using the FC model to deliver QI education was successful in a small sample of trainees.

Quality improvement (QI) tools and methodologies are essential to be taught in medical school, residency, and fellowship training programs to improve quality and safety in patient care. There is a significant need to devise new approaches to deliver higher quality care at lower costs and engage trainees in these delivery care models (1, 2).With recent emphasis from the Clinical Learning Environment Review from the Next Accreditation System of the Accreditation Council for Graduate Medical Education, and with 72% of the program directors in the United States and Canada agreeing upon the importance of QI education during training (3), most residency programs are attempting to integrate QI into the curriculum. However, incorporating a QI curriculum into a training program is challenging for various reasons including the competing schedules of other rotations, reduction in work hours, and time constraints.

We successfully incorporated QI education as a required curriculum for our fellowship trainees in 2010 (4). The development and delivery of the QI education curriculum involved two experienced Mayo Quality Academy (QA) instructors to teach and act as coaches, as well as five experienced physicians who were content experts and functioned as project champions (4). The QI education curriculum consisted of 17 weekly 90-min training sessions over a 5-month period, beginning at the start of the academic year in July (4). Instructions included didactic teaching and workshops. The content of the sessions included project discussion sessions (breakout sessions) blended with didactic classes (4). The didactic classes were designed to cover knowledge content in core QI methods including selection, initiation, and prioritization of QI projects along with appropriate conduct of a patient-outcome-driven QI project material. The breakout sessions were used to discuss the QI projects and enable greater fellow participation in the process. To accommodate the fellows’ schedules, most of the sessions were done over lunchtime and included food, which improved their ability to participate (4). All graduating pulmonary and critical care medicine (PCCM) fellows were expected to be certified through the Mayo Quality Fellows Program administered by the Mayo QA. To be certified, the fellows had to successfully pass a 25-multiple-choice-question test and conduct a QI project that needed to be approved by the Quality Review Board.

There were scheduling challenges to the program and QA leadership to successfully deliver and maintain this QI curriculum with the didactic sessions for various reasons. The didactic sessions were too long and spread weekly over a 5-month period. The trainees could not attend the sessions regularly because of their busy clinical schedule and shift-based schedules to accommodate duty-hour requirements. Also, lately the trainees were beginning to take advantage of online materials including blogs, podcasts, and social media for learning.

A recent innovation in delivering educational tools to learners consisted of creating videos as an alternative to traditional didactics, and has been popularly known as a ‘flipped classroom’ (FC) (5). In this innovative model, the didactics on videos are viewed at home on the trainees own time and schedule, and the classroom time is used to work on problem sets in a setting where they can receive help from the experts for clarifying various concepts. The potential benefits to this model includes the ability to pause, rewind, and fast-forward the content at the trainees’ own pace, and the ability of the teacher to guide the application of the knowledge in the classroom. Though this model has been used in undergraduate and graduate education, the utility and efficiency of this model in fellowship training and in delivering QI education is not known and reported.

## Methods

The objective of the FC QI education pilot was to deliver just-in-time QI didactic training in a time-efficient manner that would be effective and immediately applied to improve patient care as an integral part of the demanding PCCM fellowship curriculum. In addition, we wanted to learn about the fellows’ impressions, satisfaction, and knowledge gained by this model in order to successfully implement it as a regular component of our formal curriculum, and as a role model for other training programs to follow.

As part of the FC model, 29 videos were prepared by the Mayo QA faculty that incorporated the previously delivered 17 weekly didactic sessions that were designed to cover the silver-level [using the Applied Quality Essentials (AQE) framework] content, for a total duration of 135 min. Each video varied in length between 1 and 10 min. The overall objectives of the videos were clearly outlined and included: to articulate the case for patient-centered QI in health care and its importance; to identify, measure, and prioritize opportunities for improvement; to select and apply appropriate QI tools and methods; and to describe how to sustain long-term improvement. The video contents covered the following topics: introduction, teams, define phase, measure phase, analyze phase, intervention phase, control phase, data and measures of performance, case care and value, metric selection, six sigma, 5S, tools and methods, pull and push systems, Lean, SIPOC + R, measures and CTQ, balancing workload and built-in quality, value stream mapping, value in health care, and conclusion. Also, the videos included exercises on two common QI tools, value stream mapping and fishbone diagram.

Seven trainees participated in this FC model, that is, six PCCM fellows and one Internal Medicine resident. The FC session took place in January 2015 for 4 h. Two weeks prior to the session, the trainees were emailed a link to the videos with instructions to watch it prior to the conference, and also were provided reading materials on AQE. A reminder email was also sent 1 wk prior and 1 day prior to the conference.

The half-day session was conducted by two quality coaches from the QA and a faculty moderator. The session started with introductions followed by questions related to the videos. This was followed by case examples and a hands-on workshop on how to perform and utilize a few commonly used QI tools and methods such as value stream mapping, pareto chart, SIPOC + R, CTQ, and fishbone diagram.

The outcomes of this pilot project were to assess trainees’ satisfaction, knowledge, and skills in acquiring QI education. The outcomes were assessed by conducting surveys and taking the silver test pre-and post-session, conducted by the Mayo Quality Fellows program through the Mayo QA. The silver test contains 30 multiple-choice questions. The questions are different with the same concept in parts A and B. The pre-session silver test was on part A, while the post-session was on part B. The trainees were surveyed two times; the first survey was conducted before the start, and the second after the FC model. A five-point Likert-type scale was used for both the surveys. Research electronic data capture (REDCap survey software version 1.3.10; Vanderbilt University, 2013) was used to collect the survey data anonymously. This study was exempted by the Mayo Clinic Institutional Review Board.

## Results

All seven trainees took the pre- and post-session test; results are in Table 1. Overall improvement in the test score was 40.34% (SD 16.34) for the group, *p*<0.001.

**Table 1:** Results of the silver test to assess knowledge

Student name	Pre-intervention (%)	Post-intervention (%)
Trainee #1	47	93
Trainee #2	60	97
Trainee #3	33	90
Trainee #4	27	80
Trainee #5	70	100
Trainee #6	43	93
Trainee #7	77	87

**Fig. 1:**
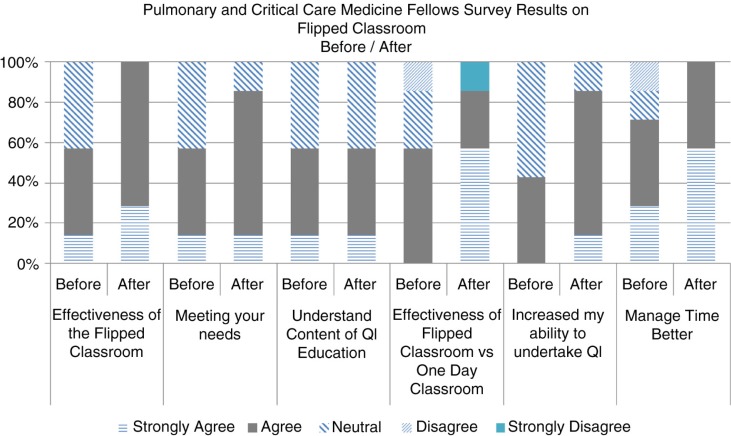
Pulmonary and critical care medicine fellows survey results on flipped classroom.

The anonymous survey results from the trainees using the Likert scale were overall positive about the FC model (Fig. 1). All seven trainees strongly agreed that we should continue with this model to deliver QI education compared to the previous format of 17 weekly sessions; and all 7 of them agreed that this was because of time efficiency, that is, the ability to view videos at their leisure and to apply those concepts in the classroom in a half-day session, compared with the previous format, and because it was an effective way to deliver QI education. Also, all seven trainees agreed that the videos were at an appropriate content, level, and pace for them to view and understand the concepts; however only 71% agreed that the videos were well-made, indicating that there is room for improvement. Fifty-five percent of the trainees agreed that the flipped model will increase their ability and confidence to undertake QI projects. Seventy-one percent of the trainees also felt that the quality coaches were effective in conducting and delivering QI education in the classroom session.

## Discussion

The Kirkpatrick validated model was used to evaluate the efficacy of the FC model to deliver QI education sessions (6). Not only was there an overall positive response to use this model to deliver future QI education sessions, the knowledge base also significantly improved using the pre- and post-session. Our pilot data in this small group of trainees clearly show that the QI education probably should be delivered using the FC model. However, innovation in a specific institute may not be successful in another institute with dissimilar contexts, resources, and needs.

We plan to use this model to deliver QI education for other fellowship programs at Mayo and will be studying the efficacy of this method in a larger sample. We will need to assess whether this model will be effective to allow the trainees to pick and complete a QI project successfully as compared to the previous format so that all graduating fellows still meet the expectation of obtaining silver certification through the Mayo Qualify Fellows Program. If results continue to be promising in a larger sample, this model can be easily disseminated to other training programs and institutions as long as the resources and needs are similar to us.
